# Evaluation of Environmental Factors Influencing Cutaneous Leishmaniasis and Importance of a Newly Designed Sand Fly (Diptera: Psychodidae) Sticky Traps

**DOI:** 10.1155/jotm/5517833

**Published:** 2026-06-17

**Authors:** Khurshaid Khan, Nazma Habib Khan, Rahaf Ajaj, Samia Al-Shouli, Nasir Ullah Shah

**Affiliations:** ^1^ Department of Zoology, Abdul Wali Khan University Mardan, Mardan, 23200, Khyber Pakhtunkhwa, Pakistan, awkum.edu.pk; ^2^ Department of Zoology, University of Peshawar, Peshawar, 25120, Khyber Pakhtunkhwa, Pakistan, uop.edu.pk; ^3^ College of Engineering, Abu Dhabi University, Abu Dhabi, 59911, UAE, adu.ac.ae; ^4^ Immunology Unit, Department of Pathology, College of Medicine, King Saud University, Riyadh, 11461, Saudi Arabia, ksu.edu.sa; ^5^ Khyber Medical University Peshawar, Peshawar, 25120, Khyber Pakhtunkhwa, Pakistan

**Keywords:** cutaneous leishmaniasis, epidemiology, Khyber Pakhtunkhwa, Pakistan, risk factors

## Abstract

Cutaneous leishmaniasis (CL) is endemic in Khyber Pakhtunkhwa (KP) Province of Pakistan, and exploring its epidemiological status is critical for its control. The current study described the spatial occurrence of CL and its associated risk factors in KP. Records of 5123 confirmed CL patients obtained from 17 district hospitals for the year 2020 revealed that Khyber district hospital had the highest influx of CL patients, followed by North Waziristan and Mardan districts. Additionally, a questionnaire to assess CL risk factors was administered to the heads of 576 randomly selected households across the same 17 districts. Multivariate logistic regression analysis of risk factors showed that education, family size greater than five individuals, information about CL, knowledge of the difference between sand flies and mosquitoes and use of insecticide sprays were risk factors for CL. Adult sand flies were sampled using sticky traps that were placed at variable heights at multiple locations within Mardan and Lower Dir districts. Collections showed that the number captured was maximum at the heights of 30–121 cm above the ground. Risk factors for CL and assessment of specific sand fly nocturnal height activities may assist in developing effective control strategies for CL in the province.

## 1. Introduction

Leishmaniasis is a vector‐borne disease affecting approximately 1.5–2.0 million people annually across 100 countries worldwide [1]. Transmission occurs through the bite of blood‐feeding phlebotomine sand flies [1–4]. The disease has three clinical forms: cutaneous leishmaniasis (CL), mucocutaneous leishmaniasis (MCL) and visceral leishmaniasis (VL) [5, 6]. The CL type predominates in the Eastern Mediterranean region (Afghanistan, Iran, Saudi Arabia and Pakistan) [7]. In Pakistan, CL is widespread in all provinces, whereas the VL form is limited to the northern areas of the country. CL is a major public health issue in Khyber Pakhtunkhwa (KP). Frequent immigration of refugees from the CL‐endemic areas of Afghanistan may contribute towards local transmission of the disease in Pakistan [8]. Reportedly, the disease is spreading its horizons to previously unaffected foci of the province [9, 10]. In KP, *Leishmania tropica*, *L. major* and, rarely, *L. infantum* are the etiological agents of CL [11].

The epidemiology of leishmaniasis is dependent on ecological and climatic factors that may be assessed using remote sensing (RS) and geographic information systems (GISs) [12]. These tools (RS and GIS) link the association between epidemiological components of the disease and environmental features to better manage the infection. Identifying risk factors is crucial for designing community‐driven approaches to control the disease [13–15].

Female sand flies biologically transmit the protozoan parasite, *Leishmania* species, the bacteria (*Bartonella bacilliformis*) and sand fly fever viruses [7]. Sand flies are active at night, and characterizing their nocturnal host‐seeking activities is crucial in eco‐epidemiological studies [16]. Sand fly flight and height are usually restricted to the ground surface, thereby avoiding wind. These insects can be collected by various methods, including sticky traps, insecticide space sprays, mouth aspiration and light traps. Among these sticky traps are interceptive rather than attractive and usually are used for determining species composition, breeding sites and nocturnal feeding behaviour [17].

CL‐infected individuals often experience disfigurement and loss of self‐esteem due to stigmatization associated with lesions on the exposed body parts. The stigmatized persons are sometimes isolated from the rest of the siblings or family to prevent further transmission of the infection. Young girls, especially with facial scars, suffer substantial psychosocial trauma since they are considered objectionable for marriage [18]. The disease is spreading from endemic to nonendemic sites, significantly challenging national and international health authorities. In KP, the government hospitals have inadequate drugs and usually have no well‐trained staff, and the victimized individuals usually buy the drugs at their own expense or travel to a dedicated CL treatment centre [19]. GIS‐based spatial analysis of cases, seasonal incidence and risk factors associated with CL in KP are not well‐documented. Vector sand fly species abundance and behaviour, specifically height activities, play a crucial role in shaping the eco‐epidemiology of CL. The present study was designed to assess the CL epidemiology in KP using multiple approaches, including retrospective (archived data), a cross‐sectional risk factors assessment for the province and sand fly height activities in selected districts.

## 2. Materials and Methods

### 2.1. Study Area

**FIGURE 1:**
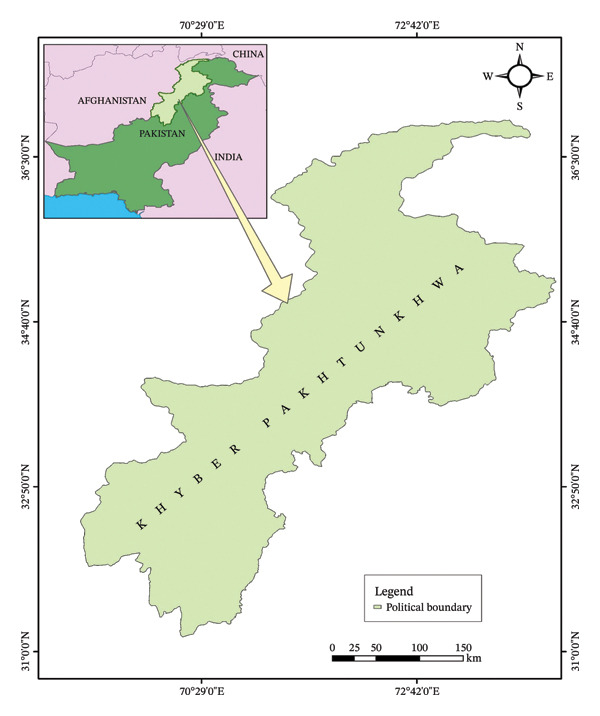
Study area map of Khyber Pakhtunkhwa, Pakistan.

The present study was carried out in the KP province of Pakistan, in the north‐western region of the country. KP shares its political borders with Afghanistan in the west, Gilgit‐Baltistan (GB) and Azad Jammu and Kashmir (AJ&K) in the north, Punjab in the east and Baluchistan in the south (Figure 1). Geographically, KP was categorized into two distinct climatic zones: the northern zone, characterized by heavy snowfall and maximum rainfall (32.2 inches per year), and the southern zone, marked by hot summers and minimal rainfall (4.5 inches per year).

### 2.2. Archived CL Data

Clinically archived CL data for 2020 were obtained from the District Headquarter Hospitals (DHQs) of Bajour, Bannu, Charsadda, Chitral, Karak, Khyber, Kohat, Lakki Marwat, Lower Dir, Malakand, Manshra, Mohmand, Mardan, North Waziristan, Nowshera, South Waziristan and Upper Dir districts. Case records included patient age, gender, nationality, locality of residence and status of the infection [i.e., new or follow‐up]. The inclusion and exclusion criteria for both fresh and follow‐up (under treatment) were based on the parasitological examination through the detection of *Leishmania* amastigotes in Giemsa‐stained skin smears. Global positioning system (GPS) coordinates (latitude and longitude) of clinically confirmed CL patients from hospital records were acquired for their locality (village) through searches via Google Earth Pro and a GPS device (Garmin eTrex, USA) in the districts.

### 2.3. Assessment of CL Risk Factors

From January–December 2020–2022, 576 houses from the abovementioned 17 districts were selected for the assessment of CL risk factors. Behavioural and sociodemographic CL risk factors were assessed through a standardized questionnaire. Household data included age, family size, socioeconomic status, vegetation in and around the house, domestic animals in the house, etc. The questionnaire was administered to the heads of randomly selected households within the study area. The questionnaire validation was based on previously published risk factors associated with CL [14, 20, 21].

### 2.4. Meteorological Data

Monthly temperature (minimum and maximum ^0^C), relative humidity (%) and rainfall (mm) 2020 data of the province were obtained from the meteorological regional office in Peshawar, Pakistan.

### 2.5. Sand Fly Collection

**FIGURE 2:**
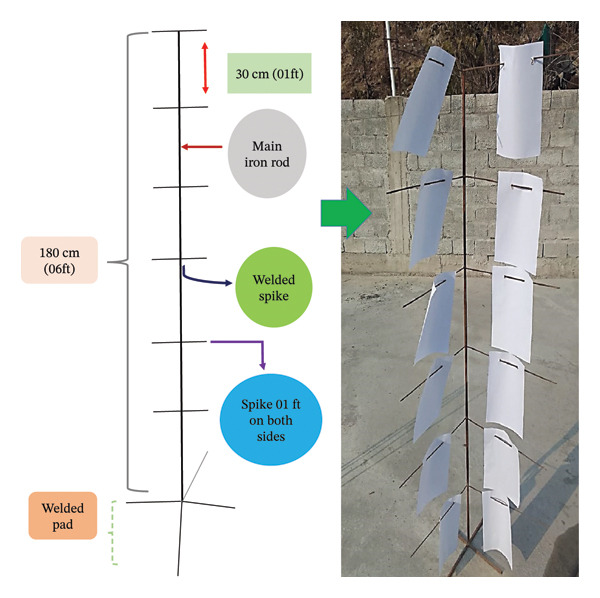
Structure of the newly designed sticky trap to detect the height pattern of sand flies.

**FIGURE 3:**
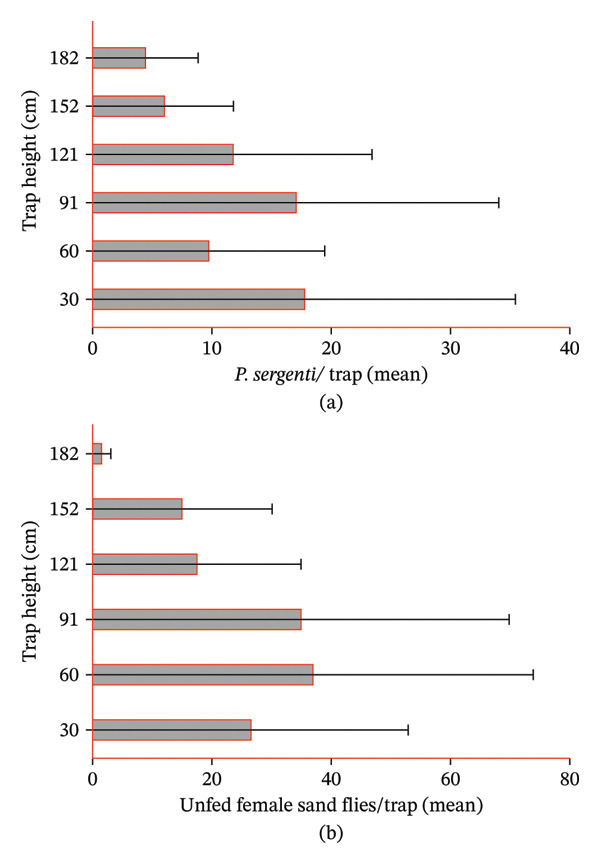
Abundance of sand flies (mean abundance per trap) in relation to height.

During the archived CL data collection, we also designed a new collection method to find out the sand flies’ specific height abundance, which has key importance in eco‐epidemiological studies of CL. Therefore, from June–August 2022, adult sand flies were sampled from Lower Dir and Mardan districts using sticky traps designed to evaluate the height of flight. A total of 10 trap stands, each 182 cm long, were fitted with six pairs of side arms, each 30 cm long, from 7 pm to 7 am (overnight) per week in domestic and peridomestic sites. Each side arm pair was separated from next by 30 cm. A total of 12 (12 × 10 = 120) sticky traps (A‐4 size white papers covered with castor oil) were installed on each stand (Figures 2 and 3). Sand flies were removed from the labelled traps and stored in 70% alcohol before further processing. In the laboratory, sand flies were dissected to expose the morphological parts and were identified using taxonomic keys [22].

### 2.6. Data Analysis

All information related to archived CL cases, GPS coordinates, sand flies and meteorological data was tabulated in Microsoft Excel for further analysis. CL patients’ cases were assessed through Spearman correlation to find out the relationship between CL cases and climatic variables using STATAv13. Analysis of sand fly species abundance with height was also conducted through Spearman correlation in STATA. Risk factors’ assessment was performed through logistic regression (univariate and multivariate) in STATAv13. The coordinate data of CL hospital records were entered into ArcGIS (Version 10.5.0; Environmental System Research Institute [ESRI], USA) for the preparation of spatial databases for CL in KP. For spatial digitization, a digital elevation model (DEM) was extracted from an advanced space‐borne thermal emission reflection radiometer (ASTER) launched in 1999 by NASA (USA) and MITI (Japan). A 12.5‐m resolution Advanced Land Observing Satellite (ALOS) phased array type L‐band synthetic aperture radar (PALSAR) DEM was obtained for the province. The KP predesigned land‐use/land‐cover, climatic and geological projection maps were obtained from the National Centre of Excellence in Geology, University of Peshawar, Pakistan. Standard categorization by the United States Geological Survey (USGS) was used for predetermined climatic, land use/land cover and geology maps of the province. Finally, the coordinates were overlaid on DEM, land‐use/land‐cover, climatic and geological maps of KP for the spatial distribution of CL cases. In ArcGIS analysis, natural break classification was used for the digitization of CL cases on the spatial maps of the province [23].

This study was approved by the Board of Studies (BoS) Committee, Department of Zoology, Abdul Wali Khan University Mardan, Pakistan.

## 3. Results

**FIGURE 4:**
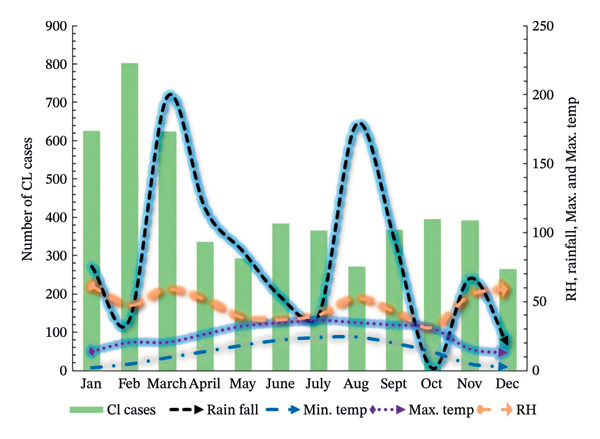
Seasonal prevalence of CL cases in Khyber Pakhtunkhwa.

A total of 5123 clinically confirmed CL case reports were obtained from 17 district hospitals of KP for 2020. The highest number of cases was reported in Khyber (*N* = 1,862, 36.35%) and North Waziristan tribal districts (*N* = 1,249, 24.38%). Males (*N* = 2,897, 56.54%) outnumbered females (*N* = 2,226, 43.45%). The majority of the CL patients presented lesions or scars on the face as compared to other body parts (Table 1). Peak number of cases (*N* = 2,052, 40.05%) were recorded in January–March, 2020, with maximum rainfall and relative humidity temperature (Figure 4). High numbers of cases were also recorded in months with the lowest temperature (minimum and maximum). Climatic variables (rainfall, temperature and relative humidity) had a negative correlation with cases, *p* value > 0.05, while a significantly negative correlation with months, *p* value < 0.05 (Table 2).

**TABLE 1:** Prevalence of archived CL cases in Khyber Pakhtunkhwa, Pakistan.

Districts	Gender		Age groups			Site of infection				Total	Prevalence (%)
	Male	Female	< 26	26–35	> 35	Face	Arm/s	Leg/s	Mixed		
Khyber	1025	837	1215	349	298	841	290	498	233	1862	36.35
NWTD^∗^	707	542	561	391	297	511	130	197	411	1249	219.42
Mardan	289	180	237	192	40	300	25	40	104	469	93.10
Lakki Marwat	251	124	264	31	80	134	24	83	134	375	7.32
Lower Dir	172	141	201	64	48	93	63	40	117	313	6.11
Upper Dir	109	113	98	71	53	90	27	31	74	222	4.33
Malakand	94	57	86	47	18	51	16	38	46	151	2.95
Bannu	65	56	55	41	25	57	17	21	27	122	2.38
Kohat	47	72	33	49	37	60	11	19	29	119	2.32
Bajaur	54	62	30	57	29	29	29	29	29	116	2.26
Charsadda	48	7	23	19	13	15	13	13	13	54	1.05
Karak	7	18	11	9	5	13	5	2	5	25	0.49
Mansehra	14	10	12	7	5	11	4	1	8	24	0.47
SWTD^∗∗^	7	1	3	2	3	3	1	3	1	8	0.16
Chitral	6	1	4	2	1	3	1	3	0	7	0.14
Mohmand	1	5	3	1	2	3	0	2	1	6	0.12
Nowshera	1	0	1	0	0	0	0	0	1	1	0.02
Subtotal	2897	2226	2837	1332	954	2214	656	1020	1233	5123	
Mean	170.41	130.94	166.88	78.35	56.12	130.24	38.59	60.00	72.53	569.22	
SD	281.25	223.49	306.86	119.19	93.54	225.78	72.16	122.46	107.97	503.77	
*r* ^*a*^	−0.75	−0.72	−0.71	−0.75	−0.71	−0.73	−0.65	−0.63	−0.77		

*Note:* *r* ^*a*^ = Spearman correlation.

Abbreviation: SD = standard deviation.

^∗^North Waziristan Tribal District.

^∗∗^South Waziristan Tribal District.

**TABLE 2:** Correlation of CL cases with climatic variables.

Variables	CL cases	*p* value	Interpretation
Rainfall	−0.09	> 0.05	Not significant
Temperature (min)	−0.18	> 0.05	Not significant
Temperature (max)	−0.03	> 0.05	Not significant
Relative humidity (RH)	−0.18	> 0.05	Not significant
Months	**−0.69^∗^**	< 0.05	Significant negative correlation

^∗^Bold value are statistically significant.

**FIGURE 5:**
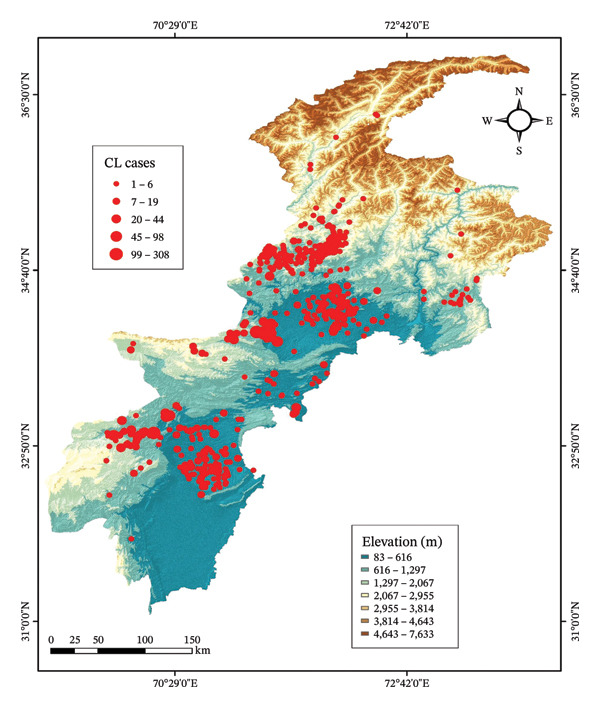
Spatial digitization of cutaneous leishmaniasis cases on DEM of KP, Pakistan.

**FIGURE 8:**
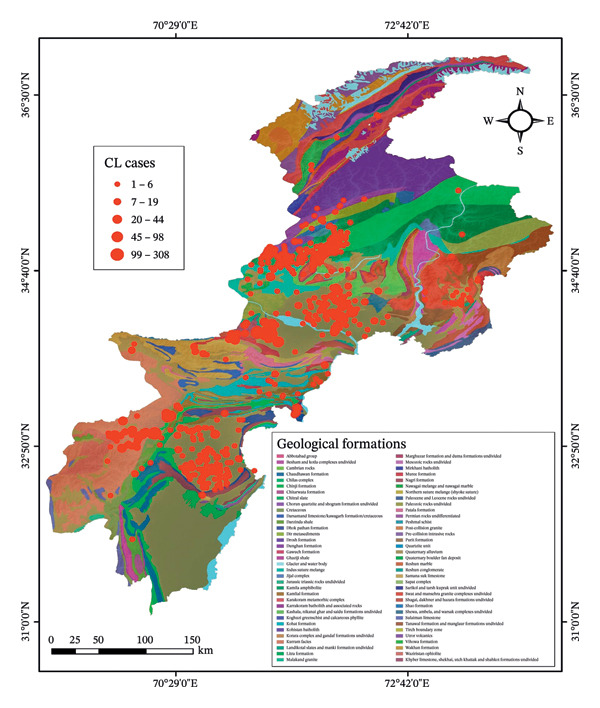
CL cased digitization on the geological map of KP.

Spatial mapping showed that the maximum number of cases was observed at 83–2,067‐m elevation (Figure 5) in agricultural land areas (Figure 6), warm semiarid climate (Figure 7) and Quaternary alluvium (QA) rock formation (Figure 8).

FIGURE 6Cutaneous leishmaniasis cases digitized on the land‐use/land‐cover map of KP, Pakistan.

**Figure:**
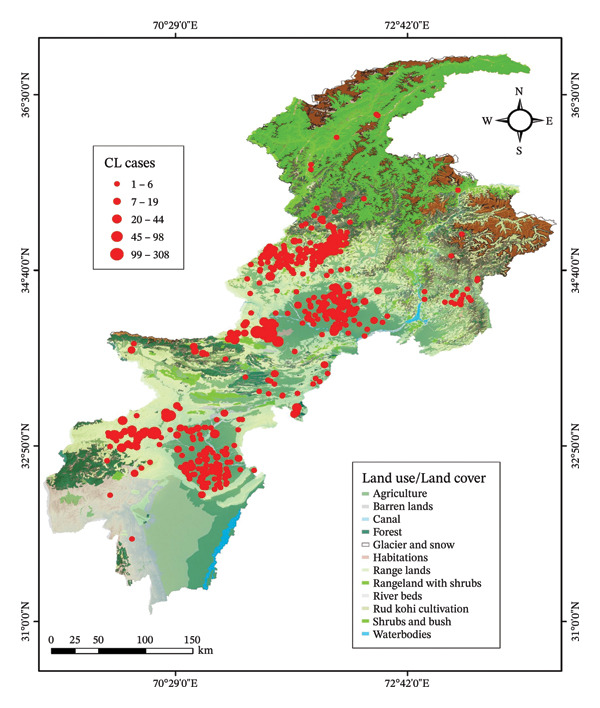

**Figure:**
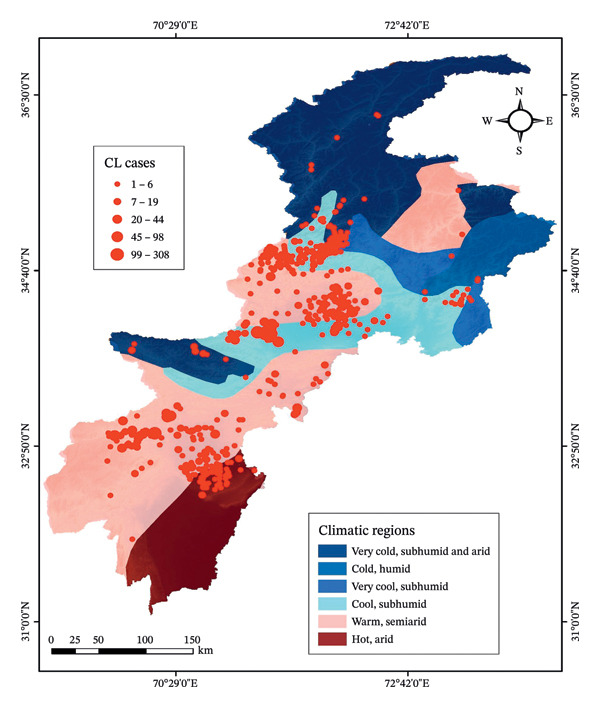

FIGURE 7CL cases digitized on the climatic map of KP.

**Figure:**
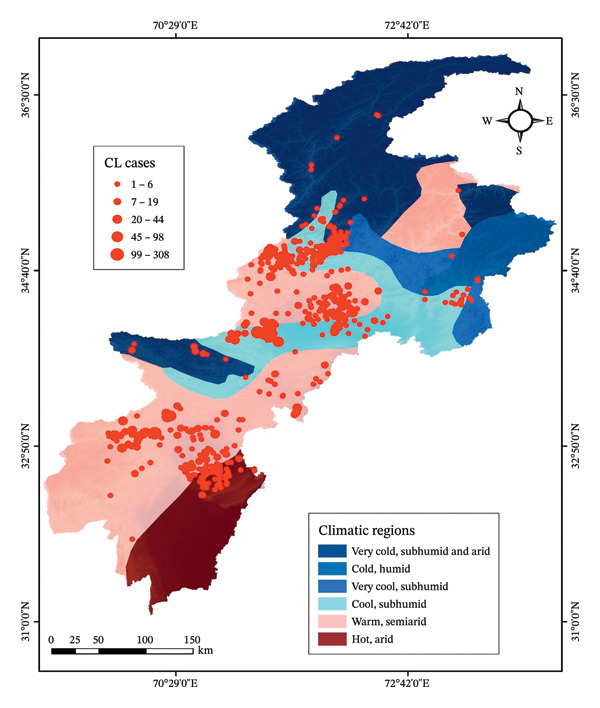

**Figure:**
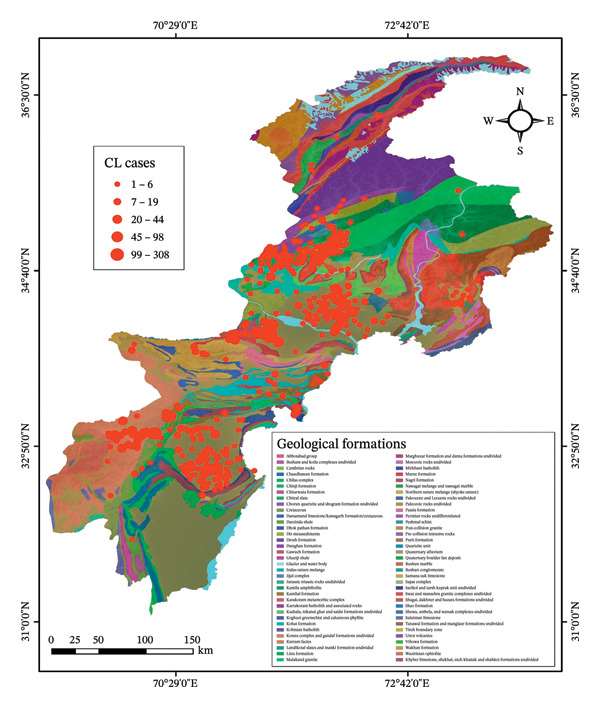

In stepwise multivariate analysis, higher education, family size (> 5 individuals), information about CL, knowledge of the difference between sand flies and mosquitoes and use of insecticide sprays increased the risk of C, *p* value < 0.005 (Table 2) (see Table 3).

**TABLE 3:** Assessment of risk factors associated with CL in Khyber Pakhtunkhwa.

Category	Number	%	Multivariate analysis	*p* value
			Odds ratio (95% confidence interval)	
Education				
Primary	177	30.73		
Secondary	128	22.22	0.527 (0.245─1.135)	0.102
Higher	271	47.05	0.380 (0.173─0.837)	< 0.005
Family size				
< 5	352	61.11		
5–15	180	30.56	3.140 (1.620─6.083)	< 0.005
> 15	44	7.12	1.939 (0.636─5.916)	0.244
Knowledge about CL				
No	431	74.83		
Yes	145	25.17	5.748 (2.980─11.085)	< 0.005
Can differentiate mosquitoes from sand flies				
No	435	75.52		
Yes	141	24.48	2.763 (1.437─5.311)	< 0.005
Use of insecticide spray				
No	215	37.33		
Yes	361	62.67	0.277 (0.143─0.536)	< 0.005

*Note:* ^∗^ *r* = Spearman correlation coefficient, bold values are statistically significant (*p* < 0 .05).

**FIGURE 9:**
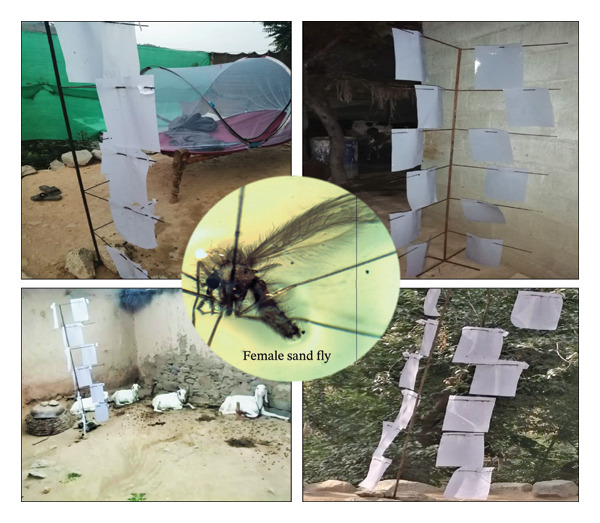
Traps of newly designed sand flies installed around human and domestic animals.

A total of 10 stands (five in each district) were installed in Lower Dir and Mardan districts (Figure 9). Overall, 987 adult sand flies comprising 6 species were collected, with *Phlebotomus sergenti*, *P. kabulensis* and *P. nuri* being the most abundant species. Most *P. sergenti* individuals were captured at heights between 30 and 121 cm above the ground; however, their abundance was negatively correlated with height (Spearman correlation, *p* value < 0.05) (Table 4).

**TABLE 4:** Correlation of sand fly species abundance with height.

Height in cm (ft)	Sand fly species							
	*P. sergenti*	*P. kabulensis*	*P. nuri*	*S. baghdadis*	*S. grekovi*	*P. papatasi*	Total	Height‐wise (%)
30 (1)	177	16	26	11	4	3	237	24.012
60 (2)	97	18	3	9	8	2	137	13.880
91 (3)	170	70	4	4	7	2	257	26.039
121 (4)	117	11	13	5	3	2	151	15.299
152 (5)	59	68	2	5	3	3	140	14.184
182 (6)	44	11	1	5	3	1	65	6.586
Subtotal	664	194	49	39	28	13	987	
Species (%)	67.275	19.656	4.965	3.951	2.837	1.317		
Mean	110.67	32.33	8.17	6.50	4.67	2.17	164.50	
SD	55.25	28.54	9.75	2.81	2.25	0.75	71.07	
^∗^ *r*	**−0.81**	0.12	−0.65	−0.78	−0.57	−0.5	−0.72	
*p* value	0.04	0.82	0.16	0.07	0.24	0.32	0.11	

*Note: ^∗^r = *Spearman correlation coefficient, bold values are statistically significant (*p* < 0 .05).

Abbreviation: SD = standard deviation.

Maximum numbers of male, unfed female and blood‐fed female sand flies were also collected at heights between 30 and 121 cm above the ground. However, unfed females were correlated negatively with height (Spearman correlation, *p* value < 0.05) (Table 5). The mean sand fly catch per trap was much higher at the lower height range compared to other heights (Figure 3).

**TABLE 5:** Gender‐wise abundance of sand flies related to stand height.

Height in cm (ft)	Feeding status of female sand flies			Males	Grand total	Height‐wise abundance (%)
	Unfed	Blood‐fed	Gravid			
30 (1)	53	4	0	180	237	24.01
60 (2)	74	0	0	63	137	13.88
91 (3)	70	0	1	186	257	26.04
121 (4)	35	1	0	115	151	15.30
152 (5)	30	4	0	106	140	14.18
182 (6)	3	0	0	62	65	6.59
Subtotal	265	9	1	712	987	
Species (%)	26.85	0.91	0.10	72.14		
Mean	44.17	1.50	0.17	118.67	164.50	
SD	26.89	1.97	0.41	54.37	71.07	
^∗^ *r*	**−0.83**	−0.19	−0.13	−0.54	−0.72	
*p* value	0.04	0.72	0.8	0.6	0.3	

## 4. Discussion

Leishmaniasis is a sand fly‐borne disease prevalent in more than 100 countries of the world, including Pakistan. However, there is no up‐to‐date registry system or spatial database for CL in the country [19]. Factors including low socioeconomic conditions, nonavailability of specialized healthcare technicians, weak immune status, lack of specific clinical laboratory facilities, limited access to health services, knowledge of vector sand flies and environmental and climatic fluctuations contribute to the disease outbreaks [24]. Sand flies, i.e., *Phlebotomus sergenti* and *P. papatasi*, were considered as suspected vector species of CL.

Archived CL data obtained from hospitals revealed that the peak number of cases was reported from Khyber, likely because Khyber Pass serves as a major strategic route for transboundary movement of Afghan refugees (susceptible to CL) between Pakistan and Afghanistan (endemic for CL) [10]. Among the other districts, the peak number of cases was recorded in the North Waziristan tribal district. Reports suggest that the disease is prevalent in the district and continues to increase progressively due to ongoing war conflicts, human displacement, presence of refugee camps and frequent migration from Afghanistan [25, 26]. This may also be attributed to limited access to basic health facilities in the tribal districts (Khyber and North Waziristan) prior to their merger with KP in 2018 [26]. However, this investigation was based on archived hospital records, and the reported cases may not reflect the true extent of the CL burden due to the self‐healing nature of the disease.

Overall, males were frequently reported to be infected with CL. In Pakistan, men are usually involved in outdoor activities and usually sleep in peridomestic sites, which may account for the greater risk of sand fly species bite [10]. Other reports suggest that males 2.5% were more likely to contract CL than females. The peak number of cases was also reported in individuals under 26 years of age, as this age group is typically involved in outdoor vocational activities. Previous studies revealed that CL cases frequently increase with age up to 15–25 years, after which the incidence declines, likely due to the development of immunity. Furthermore, age‐specific risk of CL shows that the disease is endemic in the region, as a newly introduced infection would have a substantial impact on the local communities [14, 27]. The majority of patients had a single lesion, with the face being the commonly afflicted site. Human‐exposed body parts, such as face, are predominantly prone to vector sand fly bites [20]. Several other factors such as domestic conditions, types of occupational activities and variations in sand fly species feeding behaviour are the key determinants [3, 9, 13, 14].

In KP, we additionally assessed CL risk factors through a household questionnaire in 17 districts. CL risk increased with household size (number of individuals), perhaps indicating localized CL transmission at household level [10]. Other studies also elaborated that the household clustering increases the risk of the infection, indicating a highly localized transmission at the household level by the specific sand fly species, a characteristic typically associated with *L. tropica* (causing anthroponotic cutaneous leishmaniasis [ACL]) transmission [28]. In the study factors that could be otherwise protective, including education, knowledge of CL, knowledge of the difference between sand flies and mosquitoes, and frequent use of insecticide sprays, likely increased the risk of CL in the households [24]. Thus, local communities were unable to adopt or afford protective measures despite having knowledge about the leishmaniasis and sand flies due to economic constraints and limited access to basic health facilities [3, 9, 13, 14, 24].

In spatial digitization, CL cases were plotted on maps showing elevation (DEM), climate, land use/land cover and geology of KP. CL cases were not uniformly distributed throughout the province; however, maximum numbers were observed at an elevation of 83–2,067 m. Previous reports showed the widespread occurrence of zoonotic cutaneous leishmaniasis (ZCL) at low elevation as *P. papatasi*, suspected vector sand fly species of ZCL, is frequently observed at low altitudes in the province [21, 29, 30]. Some cases were also projected at higher elevation, likely because the vector sand fly, *P. sergenti*, which transmits ACL, usually prefers high altitudes in the region [29, 31]. The maximum number of CLs was also digitized in warm and semiarid terrains, while some were highlighted in other climatic zones of the province. This may be because the vector sand flies of CL, which usually prefer diverse ecological settings, flourish in complex, dynamic environments of the region [29]. Maximum cases were elaborated in agricultural zone of the province since vegetation is known to foster the risk of the infection by providing appropriate breeding and shelters for sand flies [32]. Some cases were also highlighted in other vegetation zones, indicating that these sites also provide breeding and feeding habitats for sand flies [29]. Furthermore, CL cases peak clusters noticed in QA formation, as in eco‐epidemiological studies of CL, geological parameters play a crucial key role in disease mapping, future planning and budget allotment [32, 33].

Analysis of archived hospital records of CL patients of KP suggested a comparatively higher influx of cases in January–March of the year. Reports suggest that in the summer months (May–September), the transmission of the infection increases following the incubation period of approximately 5–6 months [33]. This is because vector sand flies show unimodal (seasonal peak abundance per year) phenology from May–September with maximum temperature, rainfall and relative humidity in the province. As these climatic factors foster sand fly activity patterns, they indirectly increase the transmission risk of CL [29, 31, 33]. Among the climatic variables, temperature and humidity have significant roles in the development of sand flies and *Leishmania* parasites. High nighttime temperatures and humidity were directly correlated with increased densities of sand fly species (*P. papatasi* and *P. sergenti*) transmitting CL [34]. Control measures such as insecticide spraying may be programmed in specific months to control sand fly populations and prevent the transmission of the disease.

Sand flies play a crucial role in the eco‐epidemiological studies of leishmaniasis, and identifying species‐specific vertical activity patterns of vector sand flies is of key importance for implementing vector control programmes [35]. Initially, we designed a sand fly height abundance study in two districts (Lower Dir and Mardan) with previously reported outbreaks of CL to find the peak activities of sand flies at specific heights. Until now, sand fly abundance at specific height ranges has not been well documented in Pakistan using this collection method. Overall, among sand fly collection techniques, sticky traps are a traditional method used worldwide for collecting sand flies [33]. However, this method has several drawbacks, including wind disturbance by wind damage caused by animals, debris accumulation from the ground and occasional refusal by residents to allow placement in indoor sites [17]. In addition, sand flies’ accurate height assessment could not be investigated through this old trapping method.

Among the collected sand fly species, *P. sergenti* and *P. papatasi* are the suspected vector species of CL in the region [29, 33]. These vector species closely coincided with the peak prevalence of CL in the region. The medical importance of other species, including *P. kabulensis, P. nuri, S. baghdadis* and *S. grekovi*, has yet to be investigated in the province. Overall, the high abundance of males and females (blood‐fed, unfed and gravid) sand flies, including *P. sergenti* and *P. papatasi* at heights of 30–121 cm above the ground, highlights maximum biting activities within this height range, which should be specifically considered in vector control programmes.

In KP, CL poses a significant public health threat, and the disease is spreading its horizons towards nonaffected areas of the province. Sociodemographic, ecological and climatic factors are the key determinants of the disease. During the study, peak aggregation of CL cases was recorded at elevations of 83 to 2,067 m in agricultural land with warm and semiarid climatic terrains of the province. Being an interceptive method, the trapping height model is strongly recommended for the nocturnally active sand fly collection, both for small and large scales, particularly in leishmaniasis endemic areas. Furthermore, dedicated health centres were recommended for the treatment of the disease to prevent further outbreaks of CL.

## Author Contributions

Conceptualization: Khurshaid Khan and Nazma Habib Khan; methodology: Khurshaid Khan and Nazma Habib Khan; validation: Khurshaid Khan and Nazma Habib Khan; formal analysis: Khurshaid Khan and Nasir Ullah Shah; investigation: Khurshaid Khan and Nasir Ullah Shah; resources: Khurshaid Khan; data curation: Khurshaid Khan; writing–original draft preparation: Khurshaid Khan; writing–review and editing: Khurshaid Khan, Nazma Habib Khan, Rahaf Ajaj and Samia Al‐Shouli; visualization: Khurshaid Khan; supervision: Khurshaid Khan; project administration: Khurshaid Khan; funding acquisition: Rahaf Ajaj.

## Funding

The authors received no financial support for the research.

## Disclosure

All authors have read and agreed to the published version of the manuscript.

## Conflicts of Interest

The authors declare no conflicts of interest.

## Data Availability

Data sharing is not applicable to this article as no datasets were generated or analysed during the current study.
